# Comparison of prognostic scores and surgical approaches to treat spinal metastatic tumors: A review of 57 cases

**DOI:** 10.1186/1749-799X-3-37

**Published:** 2008-08-28

**Authors:** Selcuk Yilmazlar, Seref Dogan, Basak Caner, Alper Turkkan, Ahmet Bekar, Ender Korfali

**Affiliations:** 1 Department of Neurosurgery, School of Medicine, Uludag University, Gorukle Kampus, Bursa 16059, Turkey

## Abstract

Surgical treatment of metastatic spinal cord compression with or without neural deficit is controversial. Karnofsky and Tokuhashi scores have been proposed for prognosis of spinal metastasis. Here, we conducted a retrospective analysis of Karnofsky and modified Tokuhashi scores in 57 consecutive patients undergoing surgery for secondary spinal metastases to evaluate the value of these scores in aiding decision making for surgery. Comparison of preoperative Karnofsky and modified Tokuhashi scores with the type of the surgical approach for each patient revealed that both scores not only reliably estimate life expectancy, but also objectively improved surgical decisions. When the general status of the patient is poor (i.e., Karnofsky score less than 40% or modified Tokuhashi score of 5 or greater), palliative treatments and radiotherapy, rather than surgery, should be considered.

## Introduction

Karnofsky and Tokuhashi scores are generally used to evaluate the life expectancy and prognosis of patients with secondary spinal metastases prior to spinal surgery for metastatic malignancy [1,3]. Spinal metastasis is associated with pain and neurological deficits, which greatly impair quality of life. For this reason, treatment of the disease is essential. Spinal metastases can extend into the epidural or intradural/intramedullary space to cause a mass effect, while vertebral metastasis can grow into the adjacent epidural space to cause pathologic fractures. Patients can suffer from severe pain even if the neural structures are not affected. Surgery, radiotherapy, or vertebroplasty, as single procedures or combined, are effective in preventing neurological deficits, stabilizing the spine, or achieving a cure [4]. Boland et al. reported that early intensive therapy can prevent spine compression and improve the quality of life of the patients [5].

Surgical approaches are generally planned according to the side of the metastasis [6]. If the anterior or middle column is affected, then an anterolateral approach would be preferred. If the posterior column is affected, then posterior approach would be chosen [7]. Combined anterior and posterior approaches would be selected if the metastasis has encircled the spinal cord. However, the side of the pathology is not the only factor affecting the surgical strategy. The type of primary pathology, the extension of the metastasis, and the general and neurologic status of the patient should also be taken into account. Therefore, standard simplified scoring systems are needed to aid decisions relating to the surgical approach and the type of the surgery. Proper selected scoring systems may predict life expectancy for spinal metastases after operation. The aim of this study compare preoperative Karnofsky and modified Tokuhashi scores with the type of the surgical approach in 57 patients with spinal metastases treated surgically and to evaluate the value of these scores in aiding decision making for surgery.

## Materials and methods

This study was conducted on 57 consecutive patients who underwent surgery for treatment of spinal metastasis at Uludag University, School of Medicine, Department of Neurosurgery from 1995 to 2005. The surgical approach to tumor resection and spinal reconstruction was determined dependent on the segments of spine involved with tumor (cervical, thoracic, lumbar, sacral), location of the tumor within the spine segment (anterior, posterior, right, left, or circumferential to neural elements).

The study population had a mean age of 48.9 ± 16.3 years and consisted of 36 males (63.1%) and 21 females (36.9%). Preoperative assessments included a medical history, a history of primary tumors, and spinal magnetic resonance imaging. All symptoms, physical findings, and neurological findings were also recorded. Following surgery, neurological assessments were performed and complications were noted. Patient survival, radiologic recurrence, and final physical and neurological states were also assessed.

Prognosis was evaluated prior to surgery using the Karnofsky performance status scale (see table 1) [8] and modified Tokuhashi scores (see table 2) [1,9]. In this modified scoring system, six parameters affecting the prognosis were scored. For the easy of analysis, all scores were categorized into the following subgroups: low risk (Karnofsky 80–100, modified Tokuhashi, 2–4 points), moderate risk (Karnofsky 50–70, modified Tokuhashi, 2–4 points), and high risk (Karnofsky 10–40, modified Tokuhashi, 5–7 points).

**Table 1:** Karnofsky performance status scale

Score	Criteria
100	Normal, no complaints, no evidence of disease
90	Able to carry on normal activity, minor signs and symptoms
80	Normal activities with effort, some signs or symptoms
70	Care for self, unable to carry on normal activity or do active work
60	Requires occasional assistance, cares for most needs
50	Requires considerable assistance and frequent care
40	Disabled, requires special care and assistance
30	Severely disabled, hospitalized, death not imminent
20	Very sick, hospitalized, active supportive care needed
10	Moribund, fatal processes are progressing rapidly
0	Dead

**Table 2:** Modified Tokuhashi scoring system for preoperative assessment of metastatic spine tumor prognosis.

Characteristics	Score
General health condition	good = 0, bad = 1
Extra-spinal bone metastasis	no = 0 , yes = 1
Other vertebral metastasis	no = 0, yes = 1
Other visceral organ metastasis	no = 0, yes = 1
Primary site of the cancer	limited = 0, diffuse = 1
Palsy	normal = 0, paresis = 1, plegia = 2

All data are expressed as mean ± standard deviation. Statistical analyses were performed using pairwise comparison of means, correlation, Pearson's test, and Fisher's exact test. A probability value less than 0.05 was considered significant.

## Results

The type of primary cancer varied among patients, with 24 (42%) having lung cancer, 8 (14%) multiple myeloma, 6 (10.5%) gastrointestinal system cancer, 4 (7.0%) non Hodgkin lymphoma, 2 (3.5%) Hodgkin lymphoma, 2 (3.5%) breast cancer, 2 (3.5%) thyroid cancer, 1 (1.8%) renal cell cancer, 1 (1.8%) testicular cancer, 1 (1.8%) ovarian cancer, 1 (1.8%) bladder cancer, and 1 (1.8%) laryngeal cancer. The primary site of cancer could not be found in four patients. In 32 (56.2%) patients, spinal metastasis was the presenting symptom, and pain was the major symptom in all of the patients. Fifty-four (94.8%) patients had neurological deficits. The metastasis was located in the cervical region in 4 (7%) patients, the thoracic region in 28 (49.2%) patients, and the lumbar region in 16 (28%) patients. Six (10.5%) patients had metastasis in the thoracolumbar junction, and 2 (3.5%) had metastasis in the servicothoracal junction. One patient (1.8%) had intramedullar metastasis.

The mean preoperative Karnofsky index was 74.2 ± 17.8 (range, 40 – 100). Thirty-two (56.1%) patients were assigned a Karnofsky index of over 80 (low risk), 22 (38.6%) patients had a Karnofsky index of 50–70 (moderate risk), and 3 (5.3%) patients had an index under 40 (low risk). The mean modified Tokuhashi score was 2.14 ± 1.19 (range, 1 – 5). The modified Tokuhashi index was 0–1 (low risk) in 25 patients (43.8%), 2–4 (moderate risk) in 31 (54.4%) patients, and 5 (high risk) in 1 (1.8%) patient. Karnofsky versus modified Tokuhashi indices were correlated in the low risk (R = 0.91), moderate risk (R = 0.99), and high risk (R = 1.00) subgroups.

An analysis of the preferred surgical approach according to Karnofsky scores revealed that, in patients with a modified Karnosky index over 80%, 15 (46.9%) patients underwent an anterolateral approach, 13 (40.6%) underwent a posterior approach, and 4 (12.5%) underwent a combined approach (see table 3). Of patients with a Karnofsky index of 50–70, 3 (13.6%) patients underwent an anterolateral approach, and 19 (86.4%) patients underwent a posterior approach. All patients with a Karnofsky score under 40 underwent a posterior approach. Of the patients with a modified Tokuhashi score of 0–1, 9 (36%) underwent an anterolateral approach, 10 (40%) a posterior approach, and 6 (24%) a combined approach (see table 4). Of those patients with a score of 2–4, 6 (19.3%) underwent an anterolateral approach and 25 (80.7%) underwent a posterior approach. The single patient with a modified Tokuhashi score of 5 underwent a posterior approach. Anterolateral, posterior, and combined approaches did not vary significantly among either modified Tokuhashi subgroups (as 0–1, 2–4, and 5–7) or Karnofsky subgroups (80–100, 50–70 and 10–40).

**Table 3:** Comparison of surgical approaches and mean survival time among Karnofsky's groups.

Karnofsky score	Surgical approach			Mean survival (months)
		
	Combined	Anterolateral	Posterior	
80–100	4	15	13	28.2 ± 16.3^§^
50–70	0	3	19	19.6 ± 12.1* ^§^
10–40	0	0	3	4.7 ± 3.6*

Patients (n)	4	18	35	

*p < 0.05, combined vs. posterior approach (Fisher's exact test)

^§^p < 0.05, posterior vs. anterolateral approach (Pearson's test)

**Table 4:** Comparison surgical approaches and mean survival time among modified Tokuhashi's groups.

Modified Tokuhashi score	Surgical approach			Mean survival (months)
		
	Combined	Anterolateal	Posterior	
0–1	6	9	10	21.4 ± 10.7* ^§^
2–4	0	6	25	11.4 ± 10.2* ^§^
5–7	0	0	1	1
Patients (n)	6	15	36	

*p < 0.05, combined vs. posterior approach (Fisher's exact test)

^§^p < 0.05, posterior vs. anterolateral approach (Pearson's test)

The mean follow up was 11.2 ± 10.4 months (range, 1 – 48 months). The mean survival time was 15.5 ± 11.5 months (range, 1 – 48 months). 6 patients (10.5%) develop local tumor recurrens at the previous level of decompression. Among the patients with a Kornofsky score over 80, the mean survival time was 28.2 ± 16.3 months. This value decreased to 19.6 ± 12.1 months in patients with a Karnofsky score of 50–70 and 4.7 ± 3.6 months in patients with a Karnofsky score under 40. Among the patients with a modified Tokuhashi score 0–1, the survival time was 21.4 ± 10.7 months. Among those with a score of 2–4, the survival time was 11.4 ± 10.2 months. The patient with a modified Tokuhashi score of 5 survived for one month. Statistical results were summarized in the tables 3 and 4.

## Discussion

Karnofsky and Tokuhashi scoring systems are currently used to determine the prognosis of the patients with metastatic spinal tumors before and after surgery [9,10]. The prognosis of spinal tumors is related to many factors such as the general condition of the patient, their ability to carry on normal activity and care for them self, and the degree of their disability. Other important factors include the presence of extraspinal bone or other organ metastasis, the histological type of the primary tumor, the limited or diffuse nature of the primary tumor, and paralysis. These prognostic factors must be taken into account for objective determination of treatment modality. This is especially true in cases of radical surgery, where the operability of the patient should be thoroughly assessed using classification systems. Therefore, in cancer patients appropriate clinical and radiological scoring methods should be chosen with determination without any delay.

Here, anterolateral and combined approaches were performed in 33.3% of patients (19/57)with Karnofsky scores of 80–100 and in 26.3% of patients (15/57) with modified Tokuhashi scores of 0–1. Posterior approach was performed in 22.8% of patients (13/57) with Karnofsky scores 80–100 and in 17.5% of patients (10/57) with modified Tokuhashi scores of 0–1. In these patients, posterior spinal cord compressions were the main component of the tumor and spinal stabilization did not required. On the other hand, posterior approaches were performed in 33.3% of patients with a Karnofsky score of 50–70 and in 43.8% of patients with modified Tokuhashi score of 2–4. Sundaresan et al stated that effective surgical treatment of neoplastic compression requires anteroposterior resection in most patients with good score to achieve the goal of total tumor resection [11]. Zarzycki D et al., also suggested that effective surgical treatment of neoplastic compression in most patients needs anteroposterior resection using instrumentation to achieve total tumor resection [12]. Thus, combined and anterolateral approaches are applied to the patients with good scores. In patients with good scores and limited lesions, as in these cases, surgery can be performed. Nevertheless, surgical modalities even in patients without any neurological deficits are still controversial, and deciding on a treatment remains difficult. According to Taneichi et al., surgery should be performed if lesions affect 50–60% of the vertebral body, since these lesions increase the risk of vertebral body collapse [13]. Additionally, lesions affecting the posterior cortex of the vertebra body and extending to the spinal cord without causing any neurological deficits carry potential risks for neurological deficits. Therefore, these lesions should be operated on even if the spinal column is stable.

For the patients without organ metastasis, the Karnofsky index may be more suitable than the Tokuhashi index for determination of treatment. However, the use of both scoring systems is most appropriate when determining treatment for spinal metastasis, especially when considering surgery. Both scoring systems separately have incapacity for determination of the clinical status of the patients. The Karnofsky scoring system is widely used for prognosis of central nervous system tumors. High Karnofsky scores are generally associated with long survival times. According to North et al., life expectancy and extended survival are highest for patients with limited pathology in one spinal segment and Karnofsky scores over 70% [14]. In accord with this report, we found that patients with Karnofsky scores of 80–100 and modified Tokuhashi scores less than 2 had the highest survival times. When deciding upon surgery, the Karnofsky score should be taken into consideration if the modified Tokuhashi score is less than 2. If the general condition is not good (Karnofsky < 40%, modified Tokuhashi > 5), then palliative treatment modalities should be considered. Tokuhashi scoring systems was suggested in estimation of early death, which can be used to predict of life expectancy for selecting surgical procedure of spinal metastases after operation [2,3,15] Radiotherapy, alone, can be used to treat patients who are not in a good general condition, which can be used to avoid major operation and are suffering pain [16,17]. Alternatively, it can be used in cases where surgery would not be effective for technical reasons [5]. Radiotherapy has been shown to be effective after surgery and can reduce pain, even if the tumor has not been totally removed [16,18].

Tumor recurrence after the surgery is one of the biggest problems associated with spinal metastasis. Nazarian et al. reported that, following surgery for spinal metastasis, recurrence was present at the same spinal level (local recurrence) in 11% of patients and at other spinal levels in 16.5% of patients [14]. In another study, local recurrence was observed in 8.4% of patients [19]. In our study, local recurrens rate was 10.5%. Palliative radiotherapy and supportive care should be considered for treatment of local recurrences without neurological deficits.

## Conclusion

In patients suffering from spinal metastasis with or without neurological deficits, surgery leads to functional recovery in low and moderate risk patients but cannot increase survival in high-risk patients. Patients in good general condition survive the longest and are good candidates for surgery, taking the vertical or horizontal extension of the tumor into consideration. The goal of the surgery should be to delay or eliminate local recurrence to prevent neurological deficits. Proper use of both modified Tokuhashi and Karnofsky scores to select surgery for patients based on life expectancy can objectively improve surgical decisions in cancer patients with spinal metastases. In future practices, comparison the results of patients with and without surgery having similar scores and comparison the predicted life expectancy and survival time may improve our treatment efforts.

## Competing interests

The authors declare that they have no competing interests.

## Authors' contributions

SY performed the case and data collection, literature review and wrote the article; SD performed literature review and helped in manuscript preparation; BC and AT helped in data and literature collection; AB and EK contributed some cases for the study. All authors read and approved the final manuscript.
